# Our Journal and the War

**Published:** 1861-07

**Authors:** 


### Our Journal and the War.

Our next issue, and perhaps during the continuance of the war, this Journal will contain reports from the army, on Surgery and Medicine. We are making arragements by which we will be able to lay before our readers reports from Surgeons in the army stationed on the seaboard and in the mountains, giving the prevalent diseases and their treatment, as well as the nature and management of surgical cases arising in the engagements that occur. The editor himself will probably be connected with the army, before the next issue, and will take every occasion to keep the readers of the Journal posted on medical matters in the army, while the editorial labors at home will be conducted by one every way competent.

